# School Absenteeism, Health-Related Quality of Life [HRQOL] and Happiness among Young Adults Aged 16–26 Years

**DOI:** 10.3390/ijerph16183321

**Published:** 2019-09-09

**Authors:** Suzanne J. van den Toren, Amy van Grieken, Wico C. Mulder, Yvonne TM Vanneste, Marjolein Lugtenberg, Marlou LA de Kroon, Siok Swan Tan, Hein Raat

**Affiliations:** 1 Department of Public Health, Erasmus University Medical Center, 3000 CA Rotterdam, The Netherlands (S.J.v.d.T.) (A.v.G.) (M.L.) (S.S.T.); 2 Dutch Center for Youth Health (NCJ), 3527 GV Utrecht, The Netherlands (W.C.M.) (Y.T.M.V.); 3 Department of Health Sciences, University Medical Center Groningen, 9700 AB Groningen, The Netherlands

**Keywords:** school absence, truancy, sickness absence, health-related quality of life, happiness, vocational education, young adults

## Abstract

This study examines the association between school absenteeism, health-related quality of life (HRQOL) and happiness among young adults aged 16–26 years attending vocational education. Cross-sectional data from a survey among 676 young adults were analyzed. School absenteeism was measured by the self-reported number of sick days in the past eight weeks and hours of truancy in the past four weeks. HRQOL was measured by the 12-item Short Form Health Survey; physical and mental component summary scores were calculated. General happiness was assessed on a scale of 0–10, higher scores indicating greater happiness. Linear regression analyses were performed. The study population had a mean age of 18.5 years (SD 2.2); 26.1% were boys. Young adults with ≥5 sick days or ≥6 h of truancy reported lower mental HRQOL compared to young adults without sickness absence or truancy (*p* < 0.05). Young adults with 1–4 and ≥5 sick days reported lower physical HRQOL compared to young adults who had not reported to be sick (*p* < 0.05). Young adults with 1–5 h and ≥6 h of truancy reported higher physical HRQOL compared to young adults who were not truant (*p* < 0.05). No associations were observed between school absence and happiness. Lower self-reported mental HRQOL was observed among young adults with more school absenteeism due to sickness or truancy. Sickness absence was additionally associated with lower physical HRQOL.

## 1. Introduction

Improving mental and physical well-being of young adults is of great importance to public health. Previous research reported an increase in the prevalence of major depressive episodes over a ten-year period among 18–24 year olds [1]. Between adolescence and young adulthood, an increase in smoking and binge drinking is demonstrated [2]. Furthermore, a study found more school absenteeism in older youth (15–17 years) compared to younger youth (12–14 years) [3]. These increases in major depressive episodes, risk (health) behaviors and school absenteeism seem to intertwine, since depression and risky health behaviors, such as tobacco use, alcohol use and risky sexual behaviors, are found to be associated with school absenteeism [3,4,5,6,7] and are clustering together [8]. It is unclear whether school absenteeism is associated with poorer health-related quality of life or with happiness. Health-related quality of life (HRQOL) is a subjective and multidimensional measure of physical functioning and well-being related to health, incorporating satisfaction with physical, social and occupational functioning, as well as vitality and psychological state of mind [9,10]. Previous studies on HRQOL in adolescents and young adults found associations with fitness and sleep quality and revealed decreased HRQOL for 16–23 years olds compared to 13–15 year olds [11,12]. Furthermore, happiness is conceptualized as both pleasure or satisfaction and the avoidance of suffering, as well as having purpose in life. Multiple factors contribute to happiness, such as education and time use and activities [13]. To our knowledge, the association between school absenteeism and HRQOL or happiness has not been studied. 

School absenteeism is divided into excused absence (e.g., sickness absence) and unexcused absence (e.g., truancy) [6]. The prevalence of school absenteeism varies greatly among and within countries. Studies reported 11% of youth truanting in the United States in the past month and 7%–48% of youth truanting in 24 European countries in the past two weeks. In The Netherlands, 13% of youth displayed truancy in the past month and 15% reported sick at least three days in the past month [14,15,16,17,18].

On the short term, school absenteeism due to health issues or truancy is associated with lower educational performance, physical complaints and psychosocial problems [3,19,20,21,22,23]. A main long-term consequence is early school leaving [6,24,25]. Youth at vocational education (i.e., education where vocational/job oriented training is offered to students) have an especially increased risk for school dropout or early school leaving; in The Netherlands in the school years 2016/2017 and 2017/2018, more than 75% of early school leaving occurred in intermediate vocational education [26]. Early school leavers are students up until the age of 23 who leave education and training without completing upper secondary education or an equivalent (i.e., attaining a basic education qualification for successfully entering the labor market) [27,28]. These non-qualified students are in a more vulnerable situation compared to peers who do obtain this qualification in terms of receiving welfare, lower earnings and reduced health [3,6,29]. Studying the association between school attendance and students’ well-being is important to support preventive intervention programs in motivating young adults to stay in school.

This study evaluates whether and how school absenteeism is associated with mental and physical HRQOL and happiness among young adults attending vocational education. The hypotheses are that young adults who are absent from school, either excused or unexcused, have lower mental and physical HRQOL and report lower rates of happiness. 

## 2. Materials and Methods 

### 2.1. Study Design

For this study, baseline data from the Medical Advice for Sick-reported Students (MASS) intervention study were used [30].

The Medical Ethics Committee of the Erasmus University Medical Centre Rotterdam reviewed the research proposal and declared that the Dutch Medical Research Involving Human Subjects Act (in Dutch: Wet medisch-wetenschappelijk onderzoek met mensen) did not apply. They issued a declaration of no objection to conduct this study and gave permission to submit the results for publication in a scientific journal in the future (proposal number MEC-2015-614).

### 2.2. Setting and Study Population

This study included youth health care providers that also provide preventive health care to students from vocational education. A total of 22 schools were contacted about the possibility to participate in the study. Ten intermediate vocational education schools in the Dutch regions of Amsterdam, West-Brabant, Utrecht and Rotterdam participated in this study. The remaining twelve schools did not have the possibility to participate because of the time investment of the research (*n* = 9), other priorities (*n* = 2) and non-response (*n* = 1). Finally, ten schools participated in the study.

**Figure 1:**
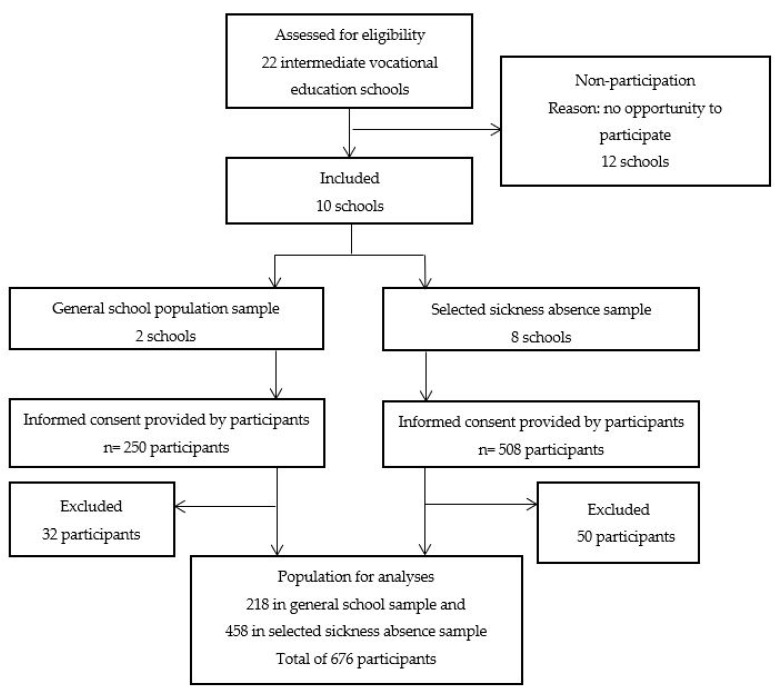
Flowchart of the study population.

Participants were students aged 16–26 years attending vocational education level one to four. The included educations were media manager/developer, assistant care, trade, interior design, nursing and technician studies (see Table 1 for detailed demographic information about the participants). Two sampling methods were used to invite students. In the first method, a selective sample was taken by an appointed coordinator or researcher. The coordinator or researcher selected students through the school sickness register. Students reporting sick at least four times in twelve weeks or more than six consecutive school days were selected. In the second method, a broader school sample was invited. Random classes were selected to participate. With this method, no selection was done based on sickness absence (see Figure 1).

Students and parents of students under 18 years of age were informed about the study through an information letter and leaflet with the informed consent form and questionnaire attached. These documents explained the aim of the study and the content of the questionnaire and included contact information of the researchers. An appointed coordinator from the participating schools sent the documents to the students and parents. Parents could object to having their child participating in the study by sending an objection letter to the school. The students were asked to provide written informed consent prior to filling out the questionnaire. The appointed coordinator collected all completed questionnaires and sent them to the researchers.

In total, 758 students provided written informed consent. For this study, we excluded students with missing information on self-reported sickness absence (*n* = 51) and on the composite scores of the HRQOL measure (*n* = 24). Twelve students gave conflicting answers on questions about their sickness absence (i.e., non-medical reasons for the absence, such as a wedding or sick family members (*n* = 12)) and three students were above the age of 26 and were excluded to limit the age range from 16–26 years (*n* = 3). Some overlap existed in the exclusion, leaving a study population of 676 participants (see Figure 1).

### 2.3. Measurements

The questionnaire contained the following topics: socio-demographic characteristics, school absenteeism, HRQOL and happiness. 

Socio-demographic characteristics of the students included gender, age, level of vocational education ranging from level one to four with one being the lowest and four being the highest level, country of birth, parents’ country of birth and living situation (i.e., living at home with caretaker).

Questions used by various national health monitors were used to measure school absenteeism [14,31]. These questions measured the duration and incidence of sickness absence in the past eight weeks as well as the reasons for sickness absence. Truancy was assessed by the question: “Have you been truanting in the past four weeks?” Response categories ranged from one (no hours) to six (more than 20 h). For analysis purpose, sickness absence was divided into three categories corresponding to the definition of chronic absence (i.e., missing 10% of school days) [32]: never, one to four days and more than four days. Truancy was also divided into three categories: never, one to five hours and more than five hours.

The 12-item Short Form Health Survey (SF-12) was used to measure self-perceived mental and physical HRQOL [33]. SF-12 includes 12 items regarding eight scales: physical functioning, role limitations due to physical problems, bodily pain, general health, vitality, social functioning, role limitation due to emotional problems and perceived mental health. Some items were recoded in order to have higher scores corresponding to better HRQOL. The raw score of each scale was transformed into 0 (the worst) to 100 (the best) before we calculated the raw Physical Component Summary (PCS-12) score and the raw Mental Component Summary (MCS-12) score. Finally, the raw PCS-12 and MCS-12 scores were transformed into the standard scores based on the normalized algorithms from the United States general population with the mean value of 50 and the standard deviation of 10. The SF-12 has been reported to have good reliability and validity [33,34].

Happiness was assessed with a single item: “Do you feel happy in general? Give an estimate of how you feel in general (so not how you feel at this moment).” Students could answer with a grade range of 0–10, with 10 being the happiest [35]. 

### 2.4. Data Analyses

Descriptive statistics were used to describe the characteristics of the participants. Chi-square tests and one-way analyses of variance were conducted to compare students with 1–4 absent days and more than four absent days to students without sickness absence in the past eight weeks. 

To examine the association of school absenteeism and socio-demographic characteristics with mental and physical HRQOL and happiness, general linear regression models were fitted for each outcome measure. First, associations were tested for school absenteeism with mental HRQOL, physical HRQOL and happiness (crude model). In the next model, socio-demographic variables (age, sex, education level, country of birth and debts) were added to the crude model as confounders (adjusted model). Additionally, interaction effects were explored between school absenteeism and socio-demographic characteristics (age, gender, country of birth and education level) for each outcome [3,11]. After applying the Bonferroni correction for multiple testing (*p* = 0.05/15 = 0.003), no statistically significant interaction was found. 

The intraclass correlation was checked due to data collection within schools as part of the MASS study. The highest intraclass correlation was observed for mental HRQOL (ICC = 0.1); therefore, we did not apply multilevel analyses.

Missing data analyses were performed by comparing students who were excluded from analyses due to missing data (*n* = 82) with included students (*n* = 676). Excluded students were significantly older (*p* = 0.03) and less often attended education level four (*p* = 0.02).

All analyses were performed using SPSS version 25 for windows (IBM Corp. Released 2017. IBM SPSS Statistics for Windows, Version 25.0, IBM Corp., Armonk, NY, USA).

## 3. Results

### 3.1. Students’ Characteristics

Table 1 shows the outcomes for school absenteeism, socio-demographic characteristics, HRQOL and happiness for three groups of sickness absence. Students were on average 18.58 years old (range 16–26), 73.8% was female and 92.1% was born in The Netherlands.

### 3.2. Mental HRQOL

The crude model in Table 2 shows the association between school absenteeism and mental HRQOL without controlling for potential confounders. The participants with more than four days of sickness absence in the past eight weeks had a significantly lower mental HRQOL compared to participants without sickness absence in the past eight weeks (B = −5.13, 95% CI = −8.84 to −1.42, *p* = 0.007). Participants with more than five hours of truancy in the past four weeks had a significantly lower mental HRQOL compared to participants who did not truant in the past four weeks (B = −4.70, 95% CI = −8.14 to −1.26, *p* = 0.007 respectively). In the adjusted model, the negative associations between school absenteeism and mental HRQOL remained (B = −5.14, 95% CI = −8.02 to −2.25, *p* = 0.001 and B = −3.84, 95% CI = −7.22 to −0.47, *p* = 0.026).

### 3.3. Physical HRQOL

The crude model in Table 3 assesses the association between school absenteeism and physical HRQOL and shows that participants with more than four days of sickness absence in the past eight weeks had a significantly lower physical HRQOL compared to participants without sickness absence −5.87 (−8.49; −3.25). In the adjusted model, participants with one to four days and more than four days of sickness absence in the past eight weeks had a lower physical HRQOL (B = −2.76, 95% CI = −4.84 to −0.74, *p* = 0.008 and B = −6.88, 95% CI = −8.95 to −4.80, *p* < 0.001) when compared to participants without sickness absence. Concurrently, participants with one to five hours and more than five hours of truancy had a higher physical HRQOL when compared to participants without truancy. 

### 3.4. Happiness

Table 4 shows that for both the crude and adjusted model, and no significant relationships were found in studying the association between school absenteeism and happiness (see Table 4). 

## 4. Discussion

This study illustrates that sickness absence and truancy are associated with mental and physical HRQOL. The hypothesized negative association between sickness absenteeism and both mental and physical HRQOL was confirmed. Furthermore, the hypothesized negative association between truancy and mental HRQOL was confirmed. In contrast to our expectations, we found a positive association between truancy and physical HRQOL. No significant associations were found between sickness absence or truancy and happiness. 

The demonstrated significant negative association especially between sickness absence and mental HRQOL in young adults warrants research and policy attention. The beta coefficient for this association showed half a standard deviation decrease in mental HRQOL and, therefore, transcends the threshold of discriminating change in HRQOL [36]. Previous reviews on the association between mental health issues and school absenteeism did not find an association between sickness absence and depression or anxiety, since mixed results from a small amount of evidence were found [4,37]. Another study did find a link between psychopathology and school absence, but did not discriminate between excused and unexcused absence [38]. Subsequently, researchers called for future research to study this association. The current research fills this gap by demonstrating a significant association between chronic sickness absence and decreased mental HRQOL. Furthermore, both students who report one to four sick days in the past eight weeks and students with more than four sick days in the past eight weeks showed decreased physical HRQOL. This is in accordance with research that found a link between physical conditions and school absenteeism [6,23]. 

Our finding that students who had been truanting showed decreased mental HRQOL is in agreement with previous reviews and studies on the association between unexcused absence and depression or anxiety [37,38,39,40]. The association between higher physical HRQOL and truancy might be explained within previous research where five out-of-school activity portfolios were identified. The highest truancy was found in the group of unstructured recreation. This group is characterized by activities such as playing non-school sports and hanging out with friends [41]. Being able to participate in these unstructured leisure time activities suggests that one is physically fit to do so, which could partly explain the improved physical HRQOL. 

We did not observe an association between school absenteeism and happiness. However, the descriptive data showed that with an increase in sick days there was a decrease in happiness. The association between happiness and school absenteeism should be further explored to establish the strength and causal pathway of the association.

When entering the three outcomes of mental and physical HRQOL and happiness in the same model, similar results were found. Sickness absence was associated with mental and physical HRQOL, and truancy was associated with mental HRQOL. The significant association between truancy and physical HRQOL disappeared in this model (data not shown).

This study has several limitations that need to be considered when interpreting the results. First, analyses comparing included participants with excluded participants due to missing data indicated that excluded participants were significantly older and less often attended a higher level of education (i.e., education level four). This could have led to selection bias if young adults with missing data were more often sick compared to included young adults. Second, students could have adhered to socially desirable answers, for example, on their amount of school absenteeism. However, we still identified associations between poor attendance and HRQOL; thus, a stronger association may be expected when objective data are used. Third, non-response bias may have occurred when young adults did not respond to the questionnaire due to their school absenteeism. The impact may, however, be limited as previous research shows that non-response in school-based health surveys is mainly linked to lifestyle factors. Physical or chronic health problems were less related to the nonparticipation [42]. Fourth, due to the cross-sectional nature of the study, the direction of the association cannot be distinguished. For example, it is not known whether low HRQOL preceded sickness absence or truancy, and vice versa. Therefore, we recommend that future studies perform longitudinal research on this topic with multiple waves to establish the causal pathway of the association. We recommend that future research study the relation between HRQOL and school absenteeism and potential mediating and confounding factors such as risk (health) behaviors, depression and relevant health indicators. Furthermore, it is recommended to study whether different reasons for the absence relate to different levels of HRQOL, i.e., if there are specific absence reasons that render the worst HRQOL scores. 

The results of the current study could raise awareness among policy makers and health care professionals working with young adults with poor school attendance. Missed days in school due to sickness or truancy seems to be associated with impaired mental HRQOL, and is therefore an area of concern. A focus on the mental health of these absent youth is necessary. It is recommended that youth health care professionals, school personnel and young adults communicate with each other about school absenteeism and collaborate to improve policy regarding school attendance and the HRQOL of young adults attending vocational education. Interventions focusing on reducing school absenteeism and improving HRQOL should be developed, implemented and improved regularly to establish maximal attendance rates and the best HRQOL for school-attending young adults. Furthermore, the most mentioned reasons for truanting indicate motivational issues, oversleeping and scheduling problems. This information might help schools to support young adults, for example, with regard to improving school schedules.

## 5. Conclusions

Students from vocational education aged 16–26 with poor school attendance due to sickness absence or truancy reported lower mental HRQOL. This underlines the importance for schools, policy makers and youth health care professionals to pay attention to the health-related quality of the lives of students who are regularly absent from school.

## Figures and Tables

**Table 1:** General characteristics, quality of life and happiness of the participants (*N* = 676).

	Total (*N* = 676)	0 Days of Sickness (*n* = 115)	1–4 Days of Sickness Absence (*n* = 275)	≥5 Days of Sickness Absence (*n* = 286)	*p*-Value
	Mean (SD)	Mean (SD)	Mean (SD)	Mean (SD)	
**School absenteeism**					**0.000**
Number of sick days in past 8 weeks	5.4 (6.3)	n/a	2.5 (1.1)	10.3 (7.1)	
*Missing n*	*0*				
	n (%)	n (%)	n (%)	n (%)	
Truancy in past 4 weeks					**0.005**
Never	412 (60.8)	85 (73.9)	161 (58.8)	165 (57.7)	
1–5 h	198 (29.2)	17 (14.8)	84 (30.7)	96 (33.6)	
≥6 h	68 (10.0)	13 (11.3)	29 (10.6)	25 (8.7)	
*Missing n*	*1*				
**Age in years**					0.527
Mean (SD)	18.5 (2.1)	18.5 (2.2)	18.5 (2.0)	18.6 (2.1)	
*Missing n*	*2*				
	n (%)	n (%)	n (%)	n (%)	
**Gender**					0.088
Boys	176 (26.1)	26 (22.8)	63 (22.9)	87 (30.4)	
Girls	499 (73.9)	88 (77.2)	212 (77.1)	199 (69.6)	
*Missing n*	*1*				
**Country of birth**					0.108
The Netherlands	*617 (92.1)*	100 (87.7)	250 (91.9)	267 (94.0)	
Other	53 (7.9)	14 (12.3)	22 (8.1)	17 (6.0)	
*Missing n*	*6*				
**Living situation**					0.064
At home with parents/caretakers	597 (88.3)	108 (93.9)	244 (88.7)	245 (85.7)	
Not at home with parents/caretakers	79 (11.7)	7 (6.1)	31 (11.3)	41 (14.3)	
*Missing n*	*0*				
**Vocational Education level**					**0.003**
Starting level and level 1	47 (7.2)	4 (3.6)	18 (6.7)	25 (9.1)	
Level 2	86 (13.1)	25 (22.7)	40 (14.9)	21 (7.6)	
Level 3	82 (12.5)	12 (10.9)	31 (11.6)	39 (14.1)	
Level 4	439 (67.1)	69 (62.7)	179 (66.8)	191 (69.2)	
*Missing n*	*25*				
**Debts**					0.101
None	543 (81.8)	95 (85.6)	229 (84.5)	219 (77.7)	
<500 euro	50 (7.5)	4 (3.6)	17 (6.3)	29 (10.3)	
>500 euro	71 (10.7)	12 (10.8)	25 (9.2)	34 (12.1)	
*Missing n*	*12*				
	Mean (SD)	Mean (SD)	Mean (SD)	Mean (SD)	
**SF-12/HRQOL**					**0.000**
Mental health composite	*45.8 (12.9)*	48.1 (11.1)	47.8 (11.1)	42.9 (14.1)	
*Missing n*	*0*				
Physical health composite	50.8 (9.3)	54.1 (6.0)	52.1 (8.1)	48.3 (10.7)	
*Missing n*	*0*				
**Grade for happiness (range 0–10)**					**0.015**
Mean (SD)	7.2 (1.6)	7.4 (1.9)	7.3 (1.5)	7.0 (1.6)	
*Missing n*	*9*				

Note: bold numbers indicate significant *p*-values.

**Table 2:** Results from the multivariable general linear models evaluating the association of school absenteeism with mental HRQOL.

SF-12 Mental Component Summary						
	Crude Model			Adjusted Model		
	B	(95% CI)	*p*-Value	B	(95% CI)	*p*-Value
**School absenteeism**						
Number of sick days in past 8 weeks						
Never	ref			ref		
1–4 days	−0.06	(−3.73; 3.62)	0.976	−0.34	(−3.19; 2.52)	0.818
≥5 days	**−5.13**	**(−8.84; −1.42)**	**0.007**	**−5.14**	**(−8.02; −2.25)**	**0.001**
Truancy in past 4 weeks						
Never	ref			ref		
1–5 h	−0.05	(−2.72; 2.61)	0.968	1.31	(−0.92; 3.55)	0.249
≥6 h	**−4.70**	**(−8.14; −1.26)**	**0.007**	**−3.84**	**(−7.22; −0.47)**	**0.026**

*Note:* bold numbers indicate significant *p*-values. Crude model: School absenteeism associated with mental HRQOL. Adjusted model: school absenteeism associated with mental HRQOL controlled for socio-demographic characteristics as possible confounders (age, gender, country of birth, living situation, education level and debts).

**Table 3:** Results from the multivariable general linear models evaluating the association of school absenteeism with physical HRQOL.

SF-12 Physical Component Summary						
	Crude Model			Adjusted Model		
	B	(95% CI)	*p*-Value	B	(95% CI)	*p*-Value
**School absenteeism**						
Number of sick days in past 8 weeks						
Never	ref			ref		
1–4 days	−0.91	(−3.51; 1.68)	0.489	**−2.70**	**(−4.74; −0.66)**	**0.010**
≥5 days	**−5.87**	**(−8.49; −3.25)**	**0.000**	**−6.86**	**(−8.93; −4.80)**	**0.000**
Truancy in past 4 weeks						
Never	ref			ref		
1–5 h	1.83	(−0.05; 3.71)	0.057	**2.98**	**(1.38; 4.58)**	**0.000**
≥6 h	1.71	(−0.72; 4.14)	0.168	**2.63**	**(0.21; 5.04)**	**0.033**

*Note:* bold numbers indicate significant *p*-values. Crude model: School absenteeism associated with physical HRQOL. Adjusted model: School absenteeism associated with physical HRQOL controlled for socio-demographic characteristics as possible confounders (age, gender, country of birth, living situation, education level and debts).

**Table 4:** Results from the multivariable general linear models evaluating the association of school absenteeism with happiness.

Happiness						
	Crude Model			Adjusted Model		
	B	(95% CI)	*p*-Value	B	(95% CI)	*p*-Value
**School absenteeism**						
Number of sick days in past 8 weeks						
Never	ref			ref		
1–4 days	0.07	(−0.41; 0.55)	0.760	−0.05	(−0.42; 0.33)	0.802
≥5 days	−0.34	(−0.83; 0.14)	0.167	−0.35	(−0.73; 0.03)	0.068
Truancy in past 4 weeks						
Never	ref			ref		
1–5 h	−0.17	(−0.52; 0.18)	0.343	0.04	(−0.25; 0.34)	0.783
≥6 h	−0.40	(−0.85; 0.04)	0.077	−0.23	(−0.67; 0.21)	0.306

Crude model: School absenteeism associated with happiness. Adjusted model: School absenteeism associated with happiness controlled for socio-demographic characteristics as possible confounders (age, gender, country of birth, living situation, education level and debts).
